# Deep Learning Approaches for Continuous Authentication Based on Activity Patterns Using Mobile Sensing

**DOI:** 10.3390/s21227519

**Published:** 2021-11-12

**Authors:** Sakorn Mekruksavanich, Anuchit Jitpattanakul

**Affiliations:** 1 Department of Computer Engineering, School of Information and Communication Technology, University of Phayao, Phayao 56000, Thailand; sakorn.me@up.ac.th; 2 Department of Mathematics, Faculty of Applied Science, King Mongkut’s University of Technology North Bangkok, Bangkok 10800, Thailand; 3 Intelligent and Nonlinear Dynamic Innovations Research Center, Science and Technology Research Institute, King Mongkut’s University of Technology North Bangkok, Bangkok 10800, Thailand

**Keywords:** continuous authentication, activity pattern, mobile sensing, deep learning, smartphone

## Abstract

Smartphones as ubiquitous gadgets are rapidly becoming more intelligent and context-aware as sensing, networking, and processing capabilities advance. These devices provide users with a comprehensive platform to undertake activities such as socializing, communicating, sending and receiving e-mails, and storing and accessing personal data at any time and from any location. Nowadays, smartphones are used to store a multitude of private and sensitive data including bank account information, personal identifiers, account passwords and credit card information. Many users remain permanently signed in and, as a result, their mobile devices are vulnerable to security and privacy risks through assaults by criminals. Passcodes, PINs, pattern locks, facial verification, and fingerprint scans are all susceptible to various assaults including smudge attacks, side-channel attacks, and shoulder-surfing attacks. To solve these issues, this research introduces a new continuous authentication framework called DeepAuthen, which identifies smartphone users based on their physical activity patterns as measured by the accelerometer, gyroscope, and magnetometer sensors on their smartphone. We conducted a series of tests on user authentication using several deep learning classifiers, including our proposed deep learning network termed DeepConvLSTM on the three benchmark datasets UCI-HAR, WISDM-HARB and HMOG. Results demonstrated that combining various motion sensor data obtained the highest accuracy and energy efficiency ratio (EER) values for binary classification. We also conducted a thorough examination of the continuous authentication outcomes, and the results supported the efficacy of our framework.

## 1. Introduction

Advances in wearable technology are progressing at incredible speeds, generating considerable interest in both professional and research communities. Smartphones, tablets, smartwatches, smart shoes, clothing, and other smart wearables are incorporating an increasing amount of extra processing capability and sensors. These augmented items serve as facilitators of ubiquitous computing, gathering data that can be utilized to offer wearers different digital solutions [1]. Numerous modern smart wearables have integrated accelerometers, gyroscopes, and magnetometers to record body motion [2,3,4]. Observing a person’s particular movement patterns may prove to be an effective tool for seamless authentication [5].

Smartphones have become the custodians of human personal data including medical data (e.g., heart rate, vaccination, and other medical treatment history), bank account information and personalized credentials for various applications and services. However, users have recently begun to express concerns about the confidentiality of their personal information as usage of smartphones increases [6,7]. Data privacy and security must be preserved [8]. Individuals often save personal identifiable information on their smartphones, while medical records, personal identification, and financial information could all be stolen. Therefore, authentication is critical to establish user identification [9,10].

The security of password-based authentication systems is largely dependent on maintaining the confidentiality of the passwords [11]. The most widely used authentication methods are still passwords, PINs, or solving a visual puzzle. While these authentication techniques have several benefits, including high accuracy, they all require the user to remember a password or a puzzle [12]. Memorizing numerous passwords or puzzles for various systems or devices (one for each device) creates a substantial burden for individuals, while previous research suggested that PINs and passwords may not provide adequate protection [13,14].

Recently, alternative biometric authentication techniques such as the face [15], voice [16], or fingerprint recognition [17] have become the norm [18]. These authentication methods do not require memorable information as they are based on a person’s unique biometric features. Nevertheless, they are still dependent on ambient circumstances. For example, facial recognition is hampered by lack of memory and processing capacity, as well as an unmanaged ambient environment. Inadequate illumination or background noise also prevent the equipment from achieving adequate facial identification. For continuous authentication, speech is processed during phone conversations. However, the speech-based authentication continues in the background, with high processing costs and consumption of battery power. Fingerprint scanning also necessitates costly sensors that the typical user does not need [19].

To respond to these challenges, motion-based biometrics provides a myriad of potential solutions that may be used to authenticate a person’s identification and, therefore, provide an additional layer of safety and theft prevention. One available option is to identify a person’s stride (walking pattern) through a collection of in-built instruments such as accelerometers [20]. Each person’s stride is unique and contains user-distinctive characteristics. Inertial sensors integrated into smartphones may be used to handle gait identification issues in security-related applications [5]. The human gait is passively visible, inconspicuous, implicit, continuous, and concurrent and can be readily assessed while users are carrying their phones [21]. While walking, the smartphone can recognize users based on their stride, allowing use of the phone’s functions without additional authentication.

Continuous authentication (CA) is a significant emerging paradigm, enabling uninterrupted real-time verification of a user’s identity, making device access as smooth as possible for authorized parties while protecting against unwanted access attempts. However, continuous user authentication is a difficult task because it involves continuously monitoring a person’s unique data, such as personality and behavioral activity characteristics. To be seamless, this management must be non-intrusive and require minimal human interaction. One potential approach is behavior pattern analysis that involves tracking the person’s physical activity pattern using inertial sensors and sending the data to classifiers trained to authenticate particular people. This method is based on the established premise that each individual has a particular activity pattern, which can be used to validate specific uniqueness. Previous research [22,23,24] used basic activity patterns such as walking and climbing and descending stairs as cognitive biometric data for continuous user authentication. Evaluating a vast number of various everyday life activities is a research gap for various reasons. One significant limitation involves determining particular actions that generate efficient biometric signatures and could be utilized independently as the foundation for a biometric system. Our research investigations contributed to the discovery of novel unique biometric activities.

This study explored continuous authentication by smartphone sensing based on diverse human activities (static, simple, and complex) to verify individual identities. The following aspects and contributions were assessed.

- DeepAuthen was developed as a continuous authentication framework for smartphones that leveraged a deep learning model to automate all user authentication phases through human activity analysis.
- DeepConvLSTM was proposed as a hybrid deep learning model that is able to capture time dependencies on features extracted by convolutional operations.
- Experimental findings demonstrated that our proposed deep learning model outperformed existing baseline deep learning approaches on three publicly available complicated datasets. The lowest energy efficiency ratios (EERs) and highest accuracies were achieved with UCI-HAR, WISDM-HARB, and HMOG datasets.

The remainder of this paper is arranged as follows: Section 2 reviews the existing literature on machine learning and deep learning methods for mobile sensing data and continuous authentication. The DeepAuthen framework for continuous authentication through mobile sensing is described in Section 3. Section 4 details the experimental design, while the findings are evaluated discussed in Section 5. Finally, Section 6 summarizes the study conclusions, including limitations and challenging future works.

## 2. Related Studies

Various studies have suggested using biometrics for continuous user authentication [22,25]. In the field of continuous authentication, inertial data are used to determine the movement, orientation, and position of a device within the surrounding environment. Methods that use this kind of data for nonintrusive authentication employ characteristics of user behavior such as gait, touchscreen operations, hand gesturing, keyboard patterns, speech, or signature movements to generate behavioral characteristics.

Zheng et al. [26] were among the first to gather an extensive continuous authentication dataset and use a one-class distance-based classifier. They combined inertial data from the device’s accelerometer and gyroscope with touchscreen, acceleration, pressure, touch area size, and time frame information between interactions. They developed study profiles of how each person held their smartphone when entering their PIN number to detect either the genuine owner or an impostor, with an EER of up to 3.6%. Trojahn et al. [27] also utilized deep learning techniques using keyboard and handwriting analytics to authenticate smartphone users based on data collected from repeatedly entering passwords. Researchers classified these images using models such as the multilayer perceptron (MLP) [28], Bayesian Net classifiers [29], and Naïve Bayes [30].

Several years later, Neverova et al. [31] utilized large-scale data from Google’s Abacus project and time-based deep feature extraction. They performed user authentication by employing recurrent neural networks (RNNs) and convolutional neural networks (CNNs). The Google Abacus dataset contains information from 1500 people who were observed in real-world settings. However, it has not been made publicly available. Researchers utilized a Dense Clockwork RNN model to classify the data. Later, Shen et al. [32] demonstrated great progress using the HMOG dataset by collecting over 27,000 data samples from ten participants including extracting wavelet, frequency, and time-domain characteristics to evaluate several algorithms including Support Vector Machines, Hidden Markov Models, and K-Nearest Neighbors.

Behavioral biometric research results are listed in Table 1, adapted from Ehatisham-ul-Haq et al. [33]. Research analyses included gestures, keystrokes, touchscreens, handwriting, speech, and locomotion. The main drawbacks of gesture-based research are that they require user interaction throughout the authentication phase. An impostor cannot be identified after the gadget is unlocked. Conversely, solutions based on keystroke dynamics have limitations and necessitate more data than other methods since they are influenced by user behavioral changes (e.g., various moods). Moreover, shifting keyboards may disturb previously acquired patterns. Touchscreen-based research also has limitations because interactions vary considerably depending on the direction, and existing user activity significantly affects interactivity. Techniques that rely on handwriting are hardly designed to enable continuous authentication because the smartphone is not stable enough to identify a change in the pattern with confidence. Background noise of the neighboring environment also has a detrimental effect on authentication technology relying on speech. Gait-based recognition is susceptible to variations in walking patterns caused by changing clothes, as well as the need to maintain the sensors on the body in a proper posture at all times [33].

Various efforts [34,35,36] have been dedicated to authenticating human activities while performing a particular task, such as entering a password, making a call, or picking up a phone from a table. A more straightforward machine learning issue may provide superior results but lacks continuity. For example, after a user is authorized, the phone does not recognize how to categorize different kinds of actions that occur until the same activity occurs repeatedly. Motion sensors can detect two types of user actions. The first is simple, while the second is complex. Walking, sitting, sleeping, ascending or descending stairs, or lying down are all examples of simple activities. By comparison, complex tasks include driving a vehicle, changing clothing, riding a bike, and exercising. Martin et al. [37] proposed a technique for detecting real-time intervals of walking, bicycling, driving, or taking the bus or train using GPS and accelerometer data. They demonstrated that GPS data achieved 96% accuracy using a random forest (RF) classifier on data processing with principal component analysis (PCA) and recursive feature elimination (RFE). By contrast, GPS remains an unfeasible option for practical implementation in CA use scenarios owing to its significant battery consumption and user authorization to execute and access data. Anguita et al. [38] used accelerometer and gyroscope data to formulate a support vector machines (SVM) model. Their model detected patterns of walking at 95% accuracy, with climbing stairs at 72%, standing at 92%, sitting at 94%, lying down at 100%, and going downstairs at 79%, while Ronao et al. [39] used comparable characteristics to investigate human activity identification using deep learning and artificial neural networks at 95% accuracy.

## 3. DeepAuthen Framework

This research study proposed DeepAuthen as a continuous authentication architecture based on deep learning using smartphone sensors. DeepAuthen was presented as a framework for continuous authentication that gathers data from smartphone sensors and feeds it to an authentication model to validate smartphone users. We investigated smartphone users’ unique use and activity patterns recorded by the sensor data used for continuous authentication.

### 3.1. Overview of the DeepAuthen Framework

**Figure 1:**
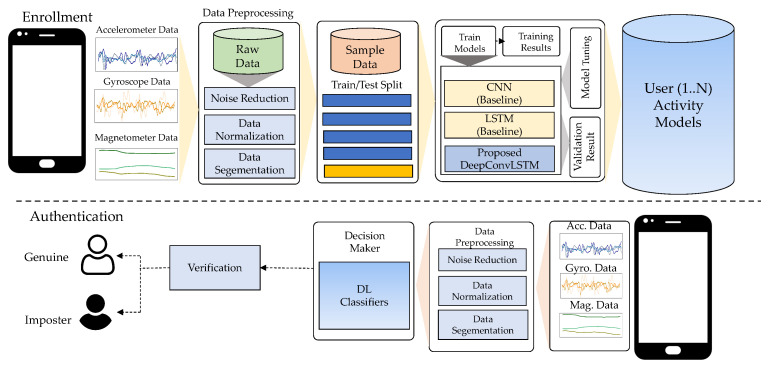
The overview of the DeepAuthen framework.

The proposed workflow of the DeepAuthen framework, shown in Figure 1, consisted primarily of four components as data acquisition, data pre-processing, data generation, and model training/testing. Data were gathered from three publicly available datasets (UCI-HAR, WISDM-HARB, and HMOG) that included static, simple, and complex human activities. The preprocessing phase segment sensor data used sliding window widths to prepare data samples for the next step. The data generation phase then separated the sample data into training and test data using a 10-fold cross-validation technique. Then, we shifted the data samples to a high-dimensional embedding space to satisfy the feature representation in our proposed DeepConvLSTM model. This process was performed using convolutional layers and a long short-term memory (LSTM) layer, as explained in the following.

### 3.2. Data Acquisition

There are many public datasets available for collecting smartphone sensor data for the purpose of human activity recognition (HAR). However, these datasets are typically insufficient for continuous authentication mechanisms because data are collected in controlled situations using fixed-mounted smartphones or released without subject information. One exception is the Human Activity Recognition using Smartphone (UCI-HAR) dataset [40], which is a collection devoted to activity recognition. The dataset has also been utilized for continuous authentication [24]. Another notable exception is the WISDM-HARB dataset, which gathers data on activity patterns to investigate activity identification and authentication using smartphone sensors. The third dataset utilized in this study was a publicly accessible dataset that gathered data specifically for continuous authentication [41], commonly referred to as the Hand Movement, Orientation, and Grasp (HMOG) dataset, generated by 100 participants throughout 24 periods using inertial and touch sensors. Participants were required to complete specified tasks throughout these periods. Each of the three datasets utilized in this study, as shown in Table 2, is described in detail as follows:

#### 3.2.1. UCI-HAR: Human Activity Recognition Using Smartphone Dataset

Various public datasets are available to gather smartphone sensors, but the UCI-HAR dataset [40] combines smartphone sensing data from 30 volunteers aged 19–48 years. In everyday life, each participant engaged in six events: walking, walking upstairs, walking downstairs, sitting, standing, and lying down. Activity data were collected using a smartphone worn around the waist. At a fixed rate of 50 Hz, the sensor data consisted of triaxial linear acceleration and triaxial angular velocity. The data were manually tagged to indicate the activity and the individual participant.

#### 3.2.2. WISDM-HARB: WISDM Human Activity Recognition and Biometric Dataset

The WISDM-HARB dataset [42] contains details from 51 participants who were required to complete 18 everyday tasks involving both simple and complex activities. For this dataset, the accelerometer and gyroscope data were collected at a steady rate of 20 Hz from smartphones and smartwatches while each participant performed these tasks for three minutes. The data were manually tagged to indicate the kind of activity and the individual participant.

This study used data from the WISDM-HARB dataset for smartphones by conducting an exploratory investigation on the sensor data. We observed that human activity data for seven individuals did not include all predefined activities and ignored the seven subjects (1616, 1618, 1637, 1638, 1639, 1640, and 1642). As a result, 44 subjects retained their smartphone data.

#### 3.2.3. HMOG: Hand Movement, Orientation, and Grasp Dataset

The HMOG has been assembled and released as publicly accessible [41]. The dataset contains accelerometer, gyroscope, and magnetometer information of tap-based characteristics such as x-y coordinates, finger-covered area, and pressure gathered from 100 smartphone users throughout 24 periods. The data were gathered in a controlled setting during several periods of smartphone use. Each period consisted of specified activities categorized as reading, writing, or map-navigation. Furthermore, each activity was conducted when both sitting and walking. Each experience was repeated four times, yielding a total of 24 experiences for each participant. The dataset contained accelerometer, gyroscope, and magnetometer sensor data, with frequency operation at 100 Hz. Each individual recorded six distinct experience patterns. Additionally, sensor information from screen interactions such as touch, keypress, scroll, pinch, and stroke were recorded, but these were irrelevant for this study.

However, specific periods were not finished owing to outlier data and missing data for some individuals in the HMOG dataset. Büch [43] determined that participant 733162 lacked accelerometer data for periods 9, 10, 11, 12, 13, and 14, while subjects 526319 and 796581 recorded only 23 periods instead of 24. As a result, we eliminated participants 526319, 796581, and 733162 from our active dataset. Li et al. [44] indicated two patients with anomalous results in HMOG but provided no additional explanations. These were presumed to be participants 526319 and 796581. The study by Centeno et al. [45] excluded ten participants from the HMOG dataset. Seven more subjects with inconsistent data were eliminated to maintain comparability with their study, while Büch’s logic [43] eliminated participants 256487, 389015, and 856401 as a result of excessive data compared to the mean, and participants 219303, 539502, 737973, and 986737 for having insufficient data.

### 3.3. Data Preprocessing

The preprocessing stage involves the preparation of raw sensor readings for subsequent steps in the proposed framework of continuous authentication. It comprises cleaning, transforming, and segmenting raw data. The cleaning step minimizes the noise using a median filter and a third-order low-pass Butterworth filter with a cutoff frequency of 20 Hz. This rate is adequate for collecting human movement since 95% of its energy is stored under 15 Hz [40]. Regarding noise filtering, the data are converted into the proper representations for the following step. In particular, the transformation step employs a Min-Max normalization method to visualize each data point’s values onto the range [0, 1]. It would support learning algorithms in balancing the impacts of different dimensions. All sensors’ normalized data are mapped to the exact size of a sliding window for the data segmentation step. In this study, we formed sensory data sequences with a duration of 2.56 s using a sliding window (128 readings for each sequence in the UCI-HAR dataset, 52 readings for each sequence in the WISDM-HARB dataset, and 256 readings for each sequence in the HMOG dataset).

**Figure 2:**
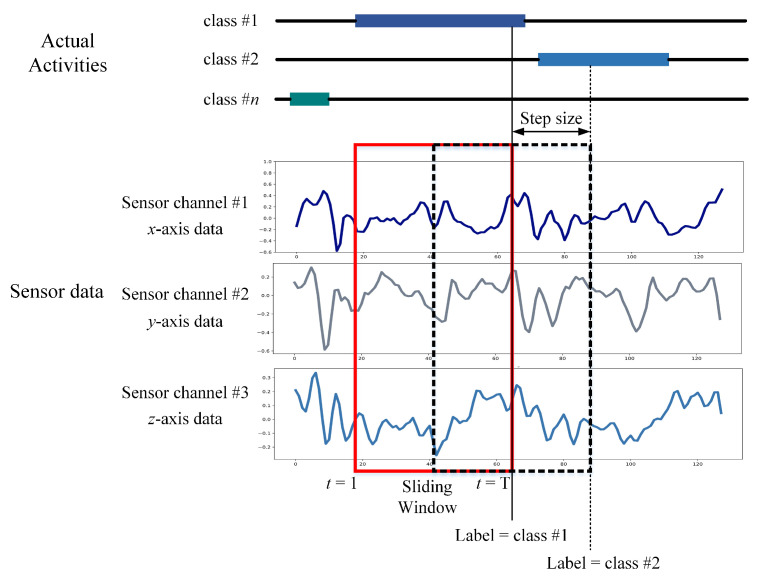
Data segmentation by the OW scheme.

In sensor-based HAR, the initial step is to generate data samples from raw sensor data. This procedure involves segmenting the raw data into identically sized short segments named temporal windows. Before training a deep learning model, raw time series data collected from wrist-worn wearable sensors is segmented into temporal segments in this study. Sliding is a commonly used method that is useful for managing flowing data. Each time interval is equal to the window size specified by Δ*t*. The $D_{t}$ denotes *X*, *Y*, and *Z* readings throughout the time interval [*t*, Δ*t*]. This study used a data segmentation technique called an overlapping temporal window (OW), which generates data samples by applying a fixed-size window to the sensor data stream. The OW technique is the most often employed in sensor-based HAR and authentication research, with a 50% overlap rate [23,24]. Nevertheless, this sample generation is considerably unbalanced since $D_{t}$ and $D_{t+1}$ share a percentage of the sensor data. Figure 2 illustrates an example of sensor data segmentation using the OW scheme. *X*, *Y*, and *Z* denote the three main components of a triaxial IMU sensor.

### 3.4. Proposed Deep Learning Network

**Figure 3:**
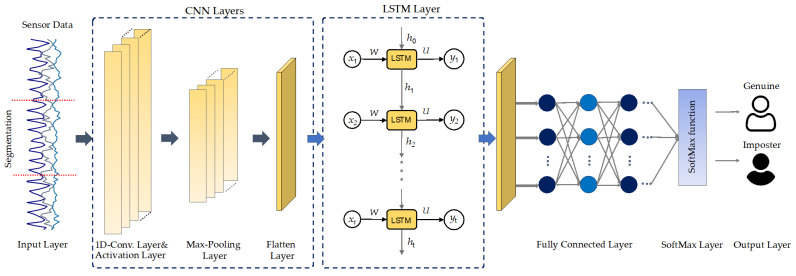
The DeepConvLSTM architecture for sensor-based continuous authentication proposed in this work.

This research addresses a deep learning model called DeepConvLSTM for continuous authentication utilizing data from smartphone sensors. Figure 3 illustrates the proposed DL network structure for continuous authentication based on activity characteristics. The proposed CA model comprises two components: the CNN network is responsible for extracting spatial characteristics from sensory input. Additionally, the LSTM network is responsible for extracting temporal dependency relations in spatial features.

Convolutional neural networks (CNNs) are commonly used deep learning models with significant feature extraction properties. The CNNs can progressively extract spatial information from the input data in an automated and efficient manner. CNNs are especially adept at processing two-dimensional image information compared to one-dimensional data such as physical or business data. The convolutional layers’ inputs are linked to the subsequent layers rather than completely connected as in conventional neural network models. Because both input sets have the same weights for subregions, the CNN’s inputs generate spatially linked outputs. However, in conventional neural network networks (NN), each input has a unique weight. Increasing the number of weights raises the input dimensionality, resulting in a more complicated network. In comparison to NN, CNN uses weight sharing and downsampling to decrease the weights and the number of connections. In the proposed model, the first convolutional layer in this study has 64 filters and a kernel size of three. The second layer has 64 filters and a kernel of size 5, whereas the pool size in the max-pooling layers has always been set to two. A flattening layer was utilized to link the convolutional and LSTM layers. The CNN subnetwork’s hyperparameters are listed in Table 3.

While CNNs are rather efficient at extracting features, they are less effective for certain classification/learning problems, including time-dependent inputs, such as the smartphone sensing data used in this work. Because the prior situation this kind of data impacts the network’s forecasts of future situations, the network would have to be aware of both the present and previous inputs. This issue could be addressed using an RNN model capable of performing classification on each aspect of a time series. The RNN describes the present input as of the previous time step and as the outcome of the prior input. The result of the RNN at time *t* is influenced by the output of the RNN at time step *t* − 1.

**Figure 4:**
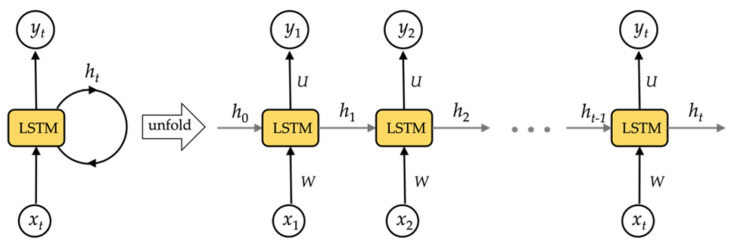
The structure of LSTM network.

RNN networks could technically be used to train for time series data of any length. In reality, RNN networks struggle from gradient disappearance when dealing with multiple time series, making it impossible to learn long-range relationships. To resolve this concern, we use a long- and short-term memory storage unit as the RNN network’s storage unit, the LSTM network. The LSTM architecture is shown in Figure 4.

**Figure 5:**
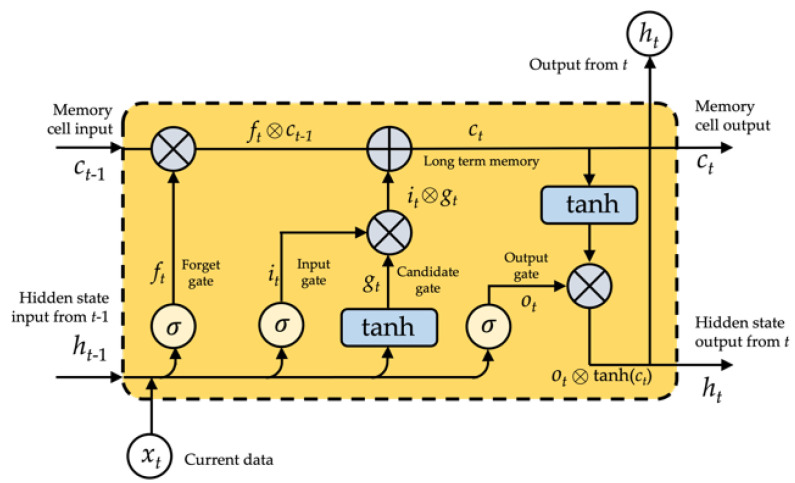
The LSTM unit.

Employing complex systems termed gates, LSTM effectively eliminates or adds information to cellular states. The gate is a technique for deliberately allowing information to flow through. It is composed of a layer of sigmoid neural networks and a pointwise multiplication function. Three gates safeguard and regulate the state of an LSTM cell: a forget gate, an input gate, and an output gate, as shown in Figure 5.

In this work, we denote an LSTM layer’s input set as *X* = {$x_{0}$, $x_{1}$, $x_{2}$, …, $x_{t}$, $x_{t+1}$, …}, its output set as *Y* = {$y_{0}$, $y_{1}$, $y_{2}$, …, $y_{t}$, $y_{t+1}$, …}, and its hidden layers as *H* = {$h_{0}$, $h_{1}$, $h_{2}$, …, $h_{t}$, $h_{t+1}$, …}. Moreover, *U*, *W*, and *V* represent the layer’s weight metrics. *U* indicates weight metrics between the input and hidden layers, *W* represents weight metrics between the hidden and hidden layers, and *V* denotes weight metrics between the hidden and output layers, respectively. The LSTM network’s computational method is as follows: the input data are converted into the hidden layer using a matrix transformation, coupled with the information from the hidden layer in the final step. Then, as shown in Figure 5, the result of the hidden layer is passed through an activation function to determine the final value of the output layer.

The hidden layer’s and output layer’s outputs could be specifically defined:

(1) $$h_{i}=\left\{\begin{array}{cc} \tanh(Ux_{i}+b_{i}^{h}) & \mathrm{while}\;i\;=\;0\\ \tanh(Ux_{i}+Wh_{i-1}+b_{i}^{h}) & \mathrm{while}\;i\;=\;1,\;2,\;3,\;\ldots \end{array}\right.$$

(2) $$y_{i}=\tanh\left(Vh_{i}+b_{i}^{y}\right)\;\;\;\mathrm{while}\;i\;=\;0,\;1,\;\ldots$$

As shown in Figure 5, the LSTM’s input gate (*i*) forget gate (*f*), output gate (*o*), and memory cell (*c*) are all intended to regulate what information should be forgotten, recalled, and updated. Gating is a technique that allows for the controlled transmission of data. It is comprised of two functions: a sigmoid and an element-wise multiplication. The output value must fall inside the range [0, 1] for multiplication to occur, allowing or preventing information flow. It is regarded best practice to calibrate these gates to 1 or near to 1 to avoid impairing training in the starting. Each parameter may be specified as follows in the LSTM unit at time *t*.

The forget gate $f_{t}$ is involved for eliminating irrelevant past information; the recurrent input $h_{t-1}\in R^{m}$ (*m* is the size of the hidden state) and current input $x_{t}\in R^{n}$ (*n* is the length of the input) are inputs to a sigmoid function σ(x) = ${\left(1+e^{-x}\right)}^{-1}$, the output $f_{t}$ is a value between 0 and 1 multiplied by the cell state ($c_{t-1}$), if $f_{t}$ equals 1, the LSTM retains this new information; alternatively, if $f_{t}$ equals 0, the LSTM discards this additional information entirely:

(3) $$f_{t}=\sigma \left(U_{f}x_{t}+W_{f}h_{t-1}+b_{f}\right)$$

The input gate $i_{t}$ is a sigmoid function, and the output is the value that is used to modify the LSTM cell:

(4) $$i_{t}=\sigma \left(U_{i}x_{t}+W_{i}h_{t-1}+b_{i}\right)$$

The state gate $g_{t}$ generates a vector of variable length. These values are added to and perform a status update:

(5) $$g_{t}=\tanh\left(U_{g}x_{t}+W_{g}h_{t-1}+b_{g}\right)$$

The output gate $o_{t}$ indicates the data from the cell state that should be produced instantaneously in combination with previously stored information:

(6) $$o_{t}=\sigma \left(U_{o}x_{t}+W_{o}h_{t-1}+b_{o}\right)$$

The update state $C_{t}$ entails forgetting what should be remembered and adding what should be added:

(7) $$c_{t}=f_{t}\;⨂\;c_{t-1}\;⨁\;i_{t}\;⨂\;g_{t}$$

The LSTM’s hidden output $h_{t}$ combines both short and long periods.

(8) $$h_{t}=o_{t}\;⨂\;\tanh\left(c_{t}\right)$$

where ⨂ denotes element-wise multiplication and ⨁ denotes element-wise addition, $W_{f}$, $W_{i}$, $W_{g}$, $W_{o}$$\in R^{m\times m}$, where *m* denotes the weighted matrices of the recurrent input $h_{t-1}$, $U_{f}$, $U_{i}$, $U_{g}$, $U_{o}$$\in R^{n\times n}$, where *n* denotes the weighted matrices of the current input $x_{t}$, $b_{i}$, $b_{f}$, $b_{c}$, $b_{o}$$\in R^{m}$.

The LSTM cell’s information processing is given in Equations (3)–(7). To beginning, there is a requirement to forget previous knowledge, which is accomplished through the forget gate. The next stage is to use an input gate to decide what additional information needs to be stored in memory. This allows for the updating of the previous cell state, $c_{t-1}$, to the new cell state, $c_{t}$. Finally, it determines which information should be sent through an output gate to the layer above.

In the proposed model, three dense layers with drop out were linked following the LSTM layer. The first dense layer has 128 neurons with a 0.25 dropout, followed by a layer of 64 neurons with a 0.25 dropout, and finally, the output layer contains two neurons. The activation function for each layer in the model is a rectified linear unit (ReLU).

The setup that performed optimally throughout the training procedure was 50 epochs with a batch size of 32. The categorical cross entropy with Adam optimizer was used as the loss function [46].

### 3.5. Performance Metrics

To evaluate an authentication scheme, the metrics are defined based on error rates required. False acceptance error (FAR) and false rejection rate (FRR) are the two primary kinds of errors. They are rising sensitivity results in an increase in FRR and a reduction in FAR. The sensitivity points at which FRR and FAR are equal error rate (EER). The following formulas illustrate some of the metrics that are used to determine authentication failures [14]. Additionally, classification measures such as accuracy and confusion matrices were utilized to assess the classification efficiency of the researched approaches [47].

#### Authentication Performance Metrics

The confusion matrix is used to quantify a classifier’s efficiency. In Table 4, we provide a confusion matrix with two potential predicted classifications: “Genuine” and “Imposter”.

- *TA* stands for True Acceptance, the number of patterns that belong to a real user and are appropriately categorized as “Genuine”.
- *TR* stands for True Reject, the number of patterns that do not belong to the real user and are appropriately categorized as “Imposter”.
- *FA* stands for False Acceptance, the number of patterns categorized as “Real” that do not belong to the genuine user.
- *FR* stands for False Reject, the number of patterns belonging to the actual user that were incorrectly categorized as “Imposter”.

The false acceptance rate (*FAR*), false reject rate (*FRR*), accuracy, and equal error rate (*ERR*) are calculated as follows using the confusion matrix:

*FAR* denotes the conditional chance that a pattern is categorized as “Genuine” if it does not already belong to it. The *FAR* is calculated using the following formula:

(9) $$FAR=\frac{FA}{FA+TR}$$

*FRR* denotes the conditional likelihood of a pattern not being classed as “Genuine” if it does belong to it. The *FRR* is calculated using the following formula:

(10) $$FRR=\frac{FR}{FR+TA}$$

Accuracy is defined as the likelihood of classifying a pattern correctly. Accuracy is defined as:

(11) $$Accuracy=\frac{TA+TR}{TA+TR+FA+FR}$$

Equal error rate (*EER*) is the error rate obtained by adjusting the system’s detection threshold to equalize *FAR* and *FRR*. The *EER* is calculated using the following formula:

(12) $$EER=\frac{FAR+FRR}{2}$$

where |FAR+FRR| is the smallest value.

## 4. Experimental Design and Evaluation of the Findings

This section discusses the experiments performed to determine the optimal effective deep learning models for continuous user authentication and the outcomes of all the studies. Our experiments were conducted on three benchmark datasets for continuous authentication using smartphone sensing data as UCI-HAR, WISDM-HARB, and HMOG. Three authentication metrics as EER, accuracy and confusion matrix were used to evaluate the deep learning models.

### 4.1. Experiment Setup

To emphasize the authentication performance of our proposed model, we compared the baseline deep learning algorithm results for continuous authentication performances of the proposed DeepConvLSTM model against a CNN model and an LSTM model. Table 5, Table 6 and Table 7 detail the hyperparameters for each model.

### 4.2. Configuration of the Environment

This study used the Google Colab Pro+ platform [48]. The Tesla V100-SXM2-16GB graphics processor module was utilized to speed deep learning model training. The DeepConvLSTM and other baseline deep learning models were implemented in Python library with Tensorflow backend (version 3.9.1) [49] and CUDA (version 8.0.6) [50]. These investigations focused on the following Python libraries:

- When reading, manipulating, and interpreting sensor data, Numpy and Pandas were utilized for data management.
- For plotting and displaying the outcomes of data discovery and model assessment, Matplotlib and Seaborn were utilized.
- Scikit-learn (Sklearn) was used in experiments as a library for sampling and data generation.
- Deep learning models were implemented and trained using TensorFlow, Keras, and TensorBoard.

### 4.3. Experimental Results

This study evaluated the proposed DeepAuthen framework against basic deep learning algorithms using three public datasets, namely UCI-HAR, WISDM-HARB, and HMOG. The following subsections provide the experimental observations of these deep learning methods trained on mobile sensing data on various datasets.

#### 4.3.1. UCI-HAR Dataset

The UCI-HAR dataset was used to gather smartphone sensor data from 30 volunteer individuals. They engaged in six activities (Wa, WU, WD, Si, St, and Ly), as stated in Table 1. As indicated in Table 8, the assessed outcomes of the deep learning models were quantified using authentication performance parameters (accuracy and EER).

**Figure 6:**
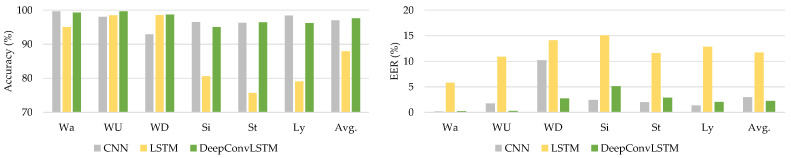
The comparative results using the UCI-HAR dataset.

Table 8 shows that the proposed DeepConvLSTM model surpasses current baseline deep learning models in average accuracy and EER through all activity patterns. Through using the UCI-HAR dataset, we analyzed our findings, which are shown in Figure 6.

#### 4.3.2. WISDM-HARB Dataset

The WISDM-HARB dataset was utilized as the second dataset in this study. This dataset contains mobile sensor data from 44 people on 18 physical activities. Table 9 summarizes the authentication performance for this dataset.

**Figure 7:**
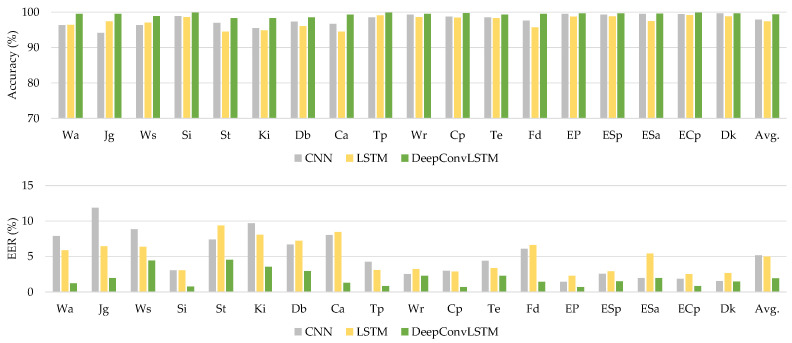
The comparative results using the WISDM-HARB dataset.

The proposed DeepConvLSTM model outperforms current baseline deep learning models in terms of average accuracy and EER for all activity patterns, as demonstrated in Table 9. Figure 7 shows the comparison findings using the WISDM-HARB dataset.

#### 4.3.3. HMOG Dataset

The HMOG dataset was utilized as the third dataset for assessing the DeepAuthen framework. This dataset comprises sensor data from 100 individuals’ smartphones relating to six different activities. Table 10 presents the experimental outcomes.

**Figure 8:**
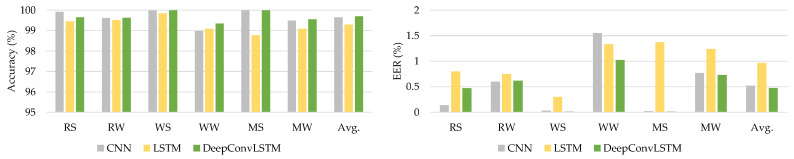
The comparative results using the HMOG dataset.

The introduced DeepConvLSTM model surpasses current baseline deep learning models in terms of average accuracy and EER for all interactions, as shown by Table 10. Figure 8 illustrates the comparison findings using the HMOG dataset.

## 5. Discussion of the Experimental Results

We analyze the research observations mentioned in Section 4 throughout this section.

### 5.1. The Effects of Different Activity Types

**Figure 9:**
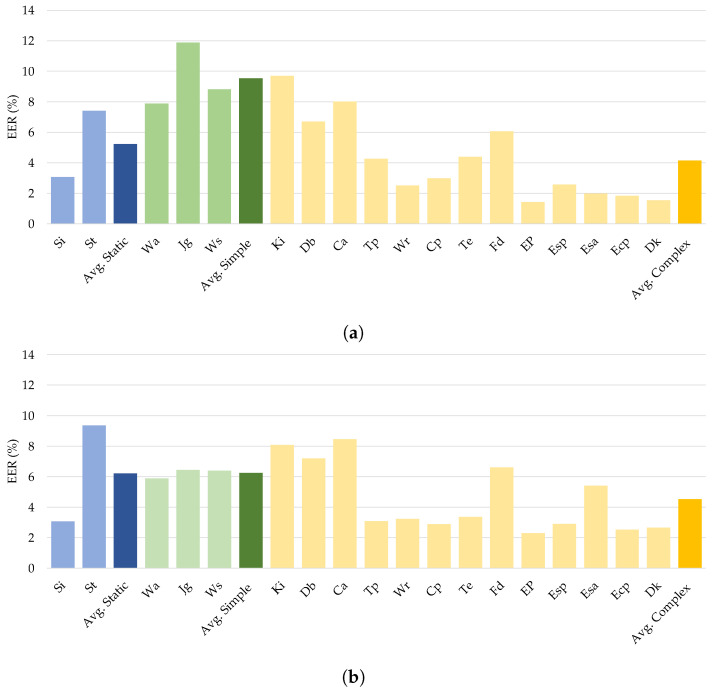

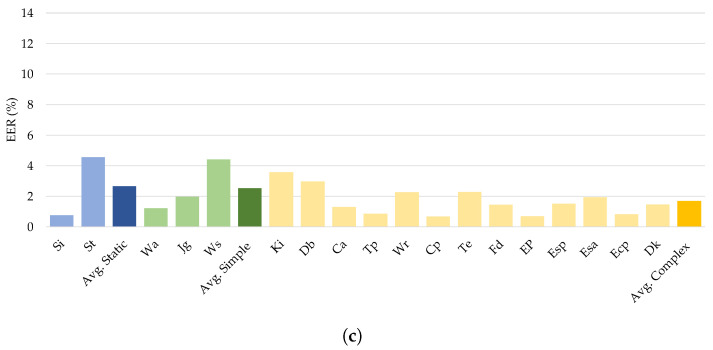
Comparison of different types of human activities on continuous authentication from (**a**) CNN, (**b**) LSTM, and (**c**) DeepConvLSTM.

Using the experimental data, we investigated the impact of different sensory inputs on authentication efficiency by evaluating continuous user authentication across the various activities. The WISDM-HARB dataset was chosen to examine the impact of activity categories since this contained three distinct kinds of activities: static, simple, and complex. The EER results in Table 9 show that all the investigated deep neural networks achieved the highest average EER when used with mobile sensing data of complex activities, as shown in Figure 9. Notably, the proposed DeepConvLSTM worked well with an average EER of 1.667% on complex mobile sensing data.

### 5.2. The Effect of Gait-Based Activity on Continuous Authentication

**Figure 10:**
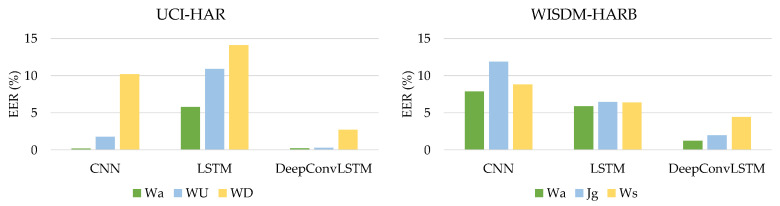
Comparison of gait-based activities of different DL classifiers on the UCI-HAR dataset.

The findings in Table 8 and Table 9 describe the performance of each classifier when applied to various gait-related tasks. Results from the two datasets UCI-HAR and WISDM-HARB included gait-related activities and indicated that optimal performance occurred when the subject moved forward in a straight line without changing direction. Consequently, the individual’s forward walking pattern was more easily identifiable than other gait-based walking patterns, as shown in Figure 10.

#### Comparison with Previous Studies

**Figure 11:**
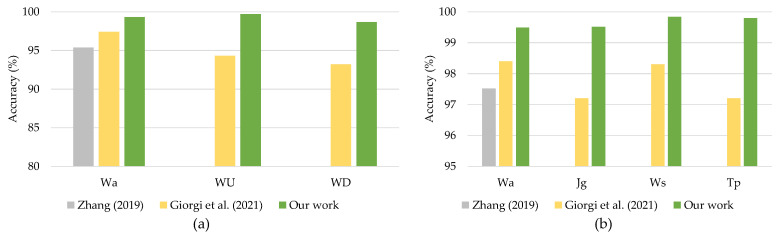
Comparison with previous works on (**a**) UCI-HAR dataset and (**b**) WISDM-HARB dataset.

For continuous authentication, the proposed DeepConvLSTM model was compared against current state-of-the-art models trained on the same datasets. Reference [23] developed an LSTM-based deep learning model to assess gait-based authentication, with smartphone sensors using the UCI-HAR and WISDM-HARB datasets. Furthermore, ref. [24] offered an LSTM autoencoder model for continuous authentication using gait analysis. These earlier studies used RNN-based deep learning models that functioned well when applied with sequencing data. Figure 11a,b compares the findings.

**Figure 12:**
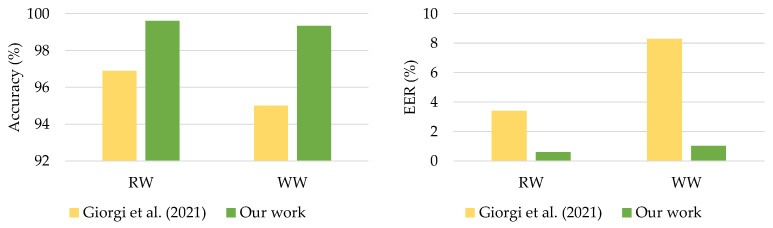
Comparison with previous works on HMOG dataset.

The comparison results presented in Figure 11 showed that the proposed DeepConvLSTM surpassed two previous studies for continuous authentication using gait-based activity data. The model improved accuracy from 1.9% to 5.47% using the UCI-HAR dataset and from 0.54% to 2.60% using the WISDM-HARB dataset. Furthermore, when compared to the HMOG dataset, the DeepConvLSTM was superior in terms of accuracy and EER [24], with results shown in Figure 12.

The DeepConvLSTM model achieved superior results due to the benefits of deep architecture based on the integration of convolutional and LSTM recurrent layers toward sensor-based authentication. Specifically, when complex activity patterns such as going upstairs, downstairs, and typing were used, the proposed DeepConvLSTM surpassed by factors of 2.6–5.47%.

## 6. Conclusions and Future Works

This section summarizes our research findings on deep learning algorithms for continuous authentication based on activity patterns identified by mobile sensing. We also discuss two significant constraints that will be addressed in a future study.

### 6.1. Conclusions

Modern smartphones have become indispensable in the activities of daily living. Our smartphones contain data to accomplish many sensitive operations such as mobile banking, communication, and storing personal pictures. As a result, demand has increased for safe authentication techniques that protect critical information from unauthorized access. This article proposed DeepAuthen as a framework for continuous authentication for smartphone users by leveraging mobile sensing data. This methodology allowed hybrid CNNs to accomplish a variety of user activity patterns by utilizing sensor data from smartphones. DeepAuthen is a simple and effective way to manage and analyze sensor data, enabling user validation without the need to engage with their devices. We conducted a series of experiments to assess DeepAuthen on the three publicly available benchmark datasets UCI-HAR, WISDM-HARB, and HMOG. Each dataset included smartphone sensing data from 30 individuals. We demonstrated that DeepAuthen could accurately authenticate users using a variety of sensors. DeepAuthen provided new state-of-the-art findings in terms of EER for continuous authentication for smartphones by utilizing sensor data.

### 6.2. Limitation and Future Works

Continuous authentication observes human activity behavior continuously. Multimodal sensors are critical for continuous authentication using behavioral biometrics. Sensors and continuous data processing are required for all operations from data collecting through to authentication and permission [51]. The implementation and use of a sufficient number of sensors improved the identification accuracy of a given action but increased the computing cost and energy consumption. Battery consumption is a practical limitation of smartphones that use a variety of proximity, light, gyroscope, barometer, accelerometer, and digital compass sensors, which are a significant drain on battery capacity. Sensory data collection requires more energy [52]. Several studies analyzed the power usage of smartphones in-depth [53,54]. In general, power management can be accomplished by turning off sensors when they are not in operation. However, continuous authentication necessitates continuous supervision and processing, and sensory power must be maintained during active sessions.

This study also addressed potential future limitations. The first issue is enhancing consumer usage of real-time authentication. This involves reducing the training time required for models. The second aspect is how to increase the efficiency of deep learning authentication models by modifying their hyperparameters. Wearable devices and the Internet of Things (IoT) are now pervasive in daily life. It is possible to trace user activities and enable implicit identification using raw data collected from different mobility sensors attached to either human bodies or things. Future research will examine the most effective and optimal approaches for utilizing raw data to design a lightweight authentication framework for individual identity verification. A critical area of future research will be to verify a method in an IoT setting where a system continually monitors or authenticates individuals as they go about their daily lives, thereby improving the accuracy and usefulness of behavior biometrics techniques.

## Figures and Tables

**Table 1:** Comparison table of our study and the related literature in term of accuracy, EER, and human activities considered.

Dataset	Work	Year	Human Activities	EER	Accuracy
UCI-HAR	Zhang [23]	2019	Walking (Wa)	-	Wa: 95.38%
	Giorgi et al. [24]	2021	Walking (Wa)	Wa: 3.1%	Wa: 97.4%
			Walking Upstairs (WU)	WU: 5.7%	WU: 94.3%
			Walking Downstairs (WD)	WD: 6.8%	WD: 93.2%
	Our work	2021	Walking (Wa)	Wa: 0.21%	Wa: 99.33%
			Walking Upstairs (WU)	WU: 0.26%	WU: 99.71%
			Walking Downstairs (WD)	WD: 2.72%	WD: 98.67%
			Sitting (Si)	Si: 5.10%	Si: 95.04%
			Standing (St)	St: 2.86%	St: 96.42%
			Laying (Ly)	Ly: 2.01%	Ly: 96.23%
WISDM-HARB	Zhang [23]	2019	Walking (Wa)	-	Wa: 97.52%
	Giorgi et al. [24]	2021	Walking (Wa)	Wa: 2.36%	Wa: 98.4%
			Jogging (Jg)	Jg: 2.6%	Jg: 97.2%
			Stairs (Ws)	Ws: 3.4%	Ws: 98.3%
			Typing (Tp)	Tp: 4.9%	Tp: 97.2%
	Our work	2021	Walking (Wa)	Wa: 1.22%	Wa: 99.49%
			Jogging (Jg)	Jg: 1.97%	Jg: 99.51%
			Stairs (Ws)	Ws: 4.41%	Ws: 99.84%
			Sitting (Si)	Si: 0.76%	Si: 99.81%
			Standing (St)	St: 4.55%	St: 98.26%
			Kicking (Ki)	Ki: 3.56%	Ki: 98.30%
			Dribbling (Db)	Db: 2.95%	Db: 98.48%
			Catch (Ca)	Ca: 1.28%	Ca: 99.25%
			Typing (Tp)	Tp: 0.84%	Tp: 99.80%
			Writing (Wr)	Wr: 2.25%	Wr: 99.50%
			Clapping (Cp)	Cp: 0.68%	Cp: 99.72%
			Teeth (Te)	Te: 2.28%	Te: 99.28%
			Folding (Fd)	Fd: 1.43%	Fd: 99.47%
			Eating Pasta (EP)	EP: 0.69%	EP: 99.66%
			Eating Soup (ESp)	ESp: 1.52%	ESp: 99.58%
			Eating Sandwich (ESa)	ESa: 1.94%	ESa: 99.55%
			Eating Chips (ECp)	ECp: 0.83%	ECp: 99.80%
			Drinking (Dk)	Dk: 1.45%	Dk: 99.60%
HMOG	Sitova et al. [22]	2015	Reading + Sitting (RS)	RW + WW: 13.72%	-
			Reading + Walking (RW)	RS + WS: 19.67%	-
			Writing + Sitting (WS)	-	-
			Writing + Walking (WW)	-	-
	Giorgi et al. [24]	2021	Reading + Walking (RW)	RW: 3.4%	RW: 96.9%
			Writing + Walking (WW)	WW: 8.3%	WW: 95%
	Our work	2021	Reading + Sitting (RS)	RS: 0.47%	RS: 99.64%
			Reading + Walking (RW)	RW: 0.62%	RW: 99.62%
			Writing + Sitting (WS)	WS: 0.01%	WS: 99.99%
			Writing + Walking (WW)	WW: 1.02%	WW: 99.34%
			Mapping + Sitting (MS)	MS: 0.01%	MS: 99.99%
			Mapping + Walking (MW)	MW: 0.73%	MW: 99.55%

**Table 2:** Characteristics of the selected datasets used to evaluate deep learning models in this work.

Dataset	Sensors	No. of Users	No. of Activities	Activities		
				Static	Simple	Complex
UCI-HAR [40]	2 Acc.	30	6	Standing	Walking	-
	1 Gyro.			Sitting	Walking Upstairs	-
				Laying	Walking Downstairs	-
WISDM-HARB [42]	1 Acc.	51	18	Sitting	Walking	Kicking
	1 Gyro.			Standing	Jogging	Dribbling
					Stairs	Catch
						Typing
						Writing
						Clapping
						Teeth
						Folding
						Eating Pasta
						Eating Soup
						Eating Sandwich
						Eating Chips
						Drinking
HMOG [41]	1 Acc.	100	6	-	Reading + Sitting	-
	1 Gyro.				Reading + Walking	
	1 Mag.				Writing + Sitting	
					Writing + Walking	
					Mapping + Sitting	
					Mapping + Walking	

**Table 3:** Characteristics of the selected datasets used to evaluate deep learning models in this work.

Layer Name	Kernel Size	Kernel Number	Padding	Stride
Conv1D${}_{1}$	3	64	2	4
Maxpooling${}_{1}$	2	None	0	1
Conv1D${}_{2}$	5	64	2	1
Maxpooling${}_{2}$	2	None	0	1

**Table 4:** Confusion matrix.

		Actual Class	
		Genuine	Imposter
**Predicted Class**	**Genuine**	*TA*	*FA*
	**Imposter**	*FR*	*TR*

**Table 5:** The summary of hyperparameters for the CNN network used in this work.

Stage	Hyperparameters		Values
Architecture	Convolution	Kernel Size	3
		Stride	1
		Filters	64
	Dropout		0.25
	Maxpooling		2
	Flatten		-
Training	Loss Function		Crossentropy
	Optimizer		Adam
	Batch Size		64
	Number of Epoches		200

**Table 6:** The summary of hyperparameters for the LSTM network used in this work.

Stage	Hyperparameters	Values
Architecture	LSTM Unit	128
	Dropout	0.25
	Dense	128
Training	Loss Function	Crossentropy
	Optimizer	Adam
	Batch Size	64
	Number of Epoches	200

**Table 7:** The summary of hyperparameters for the DeepConvLSTM network used in this work.

Stage	Hyperparameters		Values
Architecture	Convolution	Kernel Size	3
		Stride	1
		Filters	64
	Dropout		0.25
	Maxpooling		2
	Convolution	Kernel Size	5
		Stride	1
		Filters	64
	Dropout		0.25
	Maxpooling		2
	Flatten		-
	LSTM Unit		128
	Dropout		0.25
	Dense		128
Training	Loss Function		Cross-entropy
	Optimizer		Adam
	Batch Size		64
	Number of Epoches		200

**Table 8:** Average metrics on classifier evaluation of deep learning models using UCI-HAR dataset.

Activity	Authentication Performance Metrics					
	CNN		LSTM		DeepConvLSTM	
	Accuracy	EER	Accuracy	EER	Accuracy	EER
Walking	99.64% (±1.495%)	0.18% (±0.945%)	95.03% (±9.089%)	5.77% (±16.319%)	99.33% (±1.614%)	0.21% (±1.122%)
Walking Upstairs	98.06% (±3.547%)	1.77% (±3.798%)	98.51% (±1.513%)	10.90% (±18.576%)	99.71% (±1.103%)	0.26% (±1.381%)
Walking Downstairs	92.94% (±11.927%)	10.18% (±16.124%)	98.60% (±3.132%)	14.10% (±20.189%)	98.67% (±3.273%)	2.72% (±6.424%)
Sitting	96.50% (±5.359%)	2.42% (±5.729%)	80.60% (±18.519%)	15.07% (±23.740%)	95.04% (±7.160%)	5.10% (±8.307%)
Standing	96.30% (±5.193%)	1.99% (±4.138%)	75.74% (±18.921%)	11.62% (±23.066%)	96.42% (±4.789%)	2.86% (±6.516%)
Laying	98.43% (±3.066%)	1.37% (±3.839%)	79.03% (±17.076%)	12.86% (±22.946%)	96.23% (±5.387%)	2.01% (±4.672%)

**Table 9:** Average metrics on classifier evaluation of deep learning models using WISDM-HARB dataset.

Activity	Authentication Performance Metrics					
	CNN		LSTM		DeepConvLSTM	
	Accuracy	EER	Accuracy	EER	Accuracy	EER
Walking	96.28% (±4.049%)	7.88% (±9.653%)	96.42% (±5.853%)	5.88% (±9.032%)	99.49% (±1.721%)	1.22% (±3.611%)
Jogging	94.07% (±5.040%)	11.88% (±9.673%)	97.40% (±4.204%)	6.43% (±8.804%)	99.51% (±0.972%)	1.97% (±4.566%)
Stairs	96.32% (±4.480%)	8.82% (±9.797%)	97.03% (±3.799%)	6.39% (±6.667%)	98.84% (±1.343%)	4.41% (±5.172%)
Sitting	98.84% (±2.122%)	3.05% (±4.342%)	98.56% (±3.645%)	3.07% (±6.550%)	99.81% (±0.529%)	0.76% (±2.244%)
Standing	96.94% (±4.430%)	7.41% (±9.416%)	94.52% (±7.681%)	9.36% (±11.936%)	98.26% (±2.128%)	4.55% (±5.672%)
Kicking	95.47% (±5.943%)	9.70% (±12.377%)	94.82% (±7.567%)	8.08% (±10.576%)	98.30% (±4.081%)	3.56% (±8.397%)
Dribbling	97.27% (±4.190%)	6.70% (±10.039%)	95.98% (±4.861%)	7.19% (±8.618%)	98.48% (±2.287%)	2.95% (±3.899%)
Catch	96.67% (±4.863%)	8.02% (±10.829%)	94.48% (±7.139%)	8.45% (±11.730)	99.25% (±1.926%)	1.28% (±3.890%)
Typing	98.49%(±2.422%)	4.27% (±5.543%)	99.10% (±1.091%)	3.08% (±3.537%)	99.80% (±0.607%)	0.84% (±2.467%)
Writing	99.29% (±1.015%)	2.51% (±3.478%)	98.58% (±3.294%)	3.23% (±4.186%)	99.50% (±0.708%)	2.25% (±3.181%)
Clapping	98.74% (±2.806%)	2.99% (±5.510%)	98.42% (±3.548%)	2.89% (±5.300%)	99.72% (±0.723%)	0.68% (±1.714%)
Teeth	98.53% (±2.647%)	4.40% (±7.186%)	98.29% (±3.854%)	3.36% (±6.279%)	99.28% (±1.380%)	2.28% (±4.724%)
Folding	97.54% (±4.717%)	6.06% (±9.638%)	95.66% (±7.941%)	6.60% (±11.333%)	99.47% (±0.922%)	1.43% (±2.887%)
Pasta	99.49% (±1.104%)	1.43% (±2.846%)	98.73% (±2.589%)	2.29% (±3.595%)	99.66% (±1.172%)	0.69% (±1.789%)
Soup	99.29% (±1.319%)	2.56% (±4.640%)	98.79% (±2.815%)	2.91% (±6.893%)	99.58% (±1.113%)	1.52% (±4.489%)
Sandwich	99.42% (±0.837%)	1.98% (±2.630%)	97.45% (±4.477%)	5.40% (±5.831%)	99.55% (±0.900%)	1.94% (±3.882%)
Chips	99.40% (±1.124%)	1.84% (±3.329%)	99.13% (±1.800%)	2.52% (±4.715%)	99.80% (±0.530%)	0.83% (±2.254%)
Drinking	99.59% (±0.678%)	1.53% (±2.525%)	98.76% (±2.278%)	2.66% (±4.115%)	99.60% (±1.004%)	1.45% (±3.155%)

**Table 10:** Average metrics on classifier evaluation of deep learning models using HMOG dataset.

Activity	Authentication Performance Metrics					
	CNN		LSTM		DeepConvLSTM	
	Accuracy	EER	Accuracy	EER	Accuracy	EER
Read Sit	99.91% (±0.626%)	0.14% (±0.952%)	99.45% (±3.140%)	0.80% (±4.487%)	99.64% (±3.134%)	0.47% (±3.976%)
Read Walk	99.60% (±1.207%)	0.60% (±1.812%)	99.51% (±1.731%)	0.75% (±2.674%)	99.62% (±1.300%)	0.62% (±2.049%)
Write Sit	99.98% (±0.088%)	0.03% (±0.156%)	99.84% (±0.877%)	0.30% (±1.627%)	99.99% (±0.048%)	0.01% (±0.058%)
Write Walk	98.97% (±1.823%)	1.55% (±2.785%)	99.09% (±1.861%)	1.33% (±2.858%)	99.34% (±1.147%)	1.02% (±1.754%)
Map Sit	99.99% (±0.058%)	0.02% (±0.113%)	98.77% (±7.073%)	1.37% (±7.342%)	99.99% (±0.030%)	0.01% (±0.060%)
Map Walk	99.48% (±0.956%)	0.77% (±1.438%)	99.08% (±4.253%)	1.24% (±4.730%)	99.55% (±0.874%)	0.73% (±1.425%)
